# Turnover Tendency and Related Factors Among Rehabilitation Professionals in Japan: Quantifying Multiple-Choice Questionnaire Items Using Topic Analysis

**DOI:** 10.7759/cureus.82645

**Published:** 2025-04-20

**Authors:** Shinya Matsumoto, Kaori Yamaguchi, Takemi Akahane, Akira Kido, Manabu Akahane

**Affiliations:** 1 Department of Environmental Medicine and Public Health, Shimane University Faculty of Medicine, Izumo, JPN; 2 Department of Public Health, Health Management and Policy, Nara Medical University, Kashihara, JPN; 3 Department of Health and Welfare Services, National Institute of Public Health, Wako, JPN; 4 Department of Gastroenterology, Nara Medical University, Kashihara, JPN; 5 Department of Rehabilitation Medicine, Nara Medical University, Kashihara, JPN

**Keywords:** internet panel survey, occupational therapist, physical therapist, retention, topic model analysis

## Abstract

Background

Rehabilitation needs have recently increased globally as societies age. Preventing well-trained rehabilitation professional turnover is important to provide effective treatments and minimize losses. However, reasons for turnover among rehabilitation professionals have not been reported in Japan.

Objective

This study aimed to clarify the factors related to turnover intention among rehabilitation professionals in Japan.

Methods

We conducted a cross-sectional internet panel survey of 515 physical and occupational therapists. Turnover intention and potential items relevant to turnover were measured using a rating scale and multiple-choice questions. We performed a two-step analysis: topic analysis and factor analysis. Topic analysis on multiple-choice questions extracted the topics described in the text. Factor analysis was consequently performed, which extracted each respondent's motivations (topics) and the results of the graded evaluation method. Linear regressions were performed using the resultant factors and the responses regarding turnover intention.

Results

Four factors were significantly relevant to turnover intention (p*<*0.05): “People with low job satisfaction are more likely to quit”; “People will quit because of an unbalanced relationship between working hours and salary”; “Relationships among coworkers are important”; and “People with high motivation for research activities are more likely to quit.”

Conclusions

Our results clarified that the factors relevant to turnover among rehabilitation professionals in Japan are common to health professionals in other countries. Increasing job satisfaction and building a working environment with good relationships among coworkers might prevent turnover among rehabilitation professionals.

## Introduction

Recently, rehabilitation needs have increased globally because of the demographic transition to aging societies in many countries [1,2]. A team approach to rehabilitation is effective in acute and post-acute phases when medical rehabilitation is most intensively provided [3,4]. Strasser et al. [3] reported that team functions such as communication, utility of quality information, order and organization, and task orientation are associated with patient outcomes in rehabilitation for stroke. Systematic reviews regarding rehabilitation for brain injury [4] demonstrate the effectiveness of a multi-disciplinary team approach. A relationship between team maturation, such as strengthening communication, and improved patient outcomes has also been reported [5-7]. Furthermore, on-the-job training for professionals, which is costly, is essential for team building [6-8]. Therefore, retaining well-trained therapists is important for effective rehabilitation; therapist turnover could negatively impact hospitals or other medical facilities.

Losses due to health professional turnover have been reported in the field of nursing. Nurse turnover has led to financial loss in some countries [9-11]. For instance, the cost per registered nurse turnover represents half an average salary, which mostly compensates for temporary cover [10]. Nurse turnover also leads to non-financial losses. Bae et al. [12] showed that a lower turnover rate is associated with higher patient satisfaction, fewer patient falls, and fewer medication errors. Reilly et al. [13] demonstrated a negative relationship between nurse turnover and patient satisfaction. Park et al. [14] reported that increased nurse turnover is associated with an increased prevalence of pressure ulcers among their patients. Regarding healthcare professionals other than nurses, Zhao et al. [15] reported a positive relationship between the staff turnover ratio and the cost per consultation on primary care in remote Australia. Preventing professional turnover is crucial to providing substantial rehabilitation services and minimizing losses.

Studies on nurse turnover are abundant [16-20]. However, reports on turnover among rehabilitation professionals, such as physical therapists (PTs) and occupational therapists (OTs), remain limited. Studies conducted in the United States, Australia, and South Korea identify salary and work stress as factors contributing to turnover intention among rehabilitation professionals [21-24]. However, the factors related to turnover could vary due to contextual differences between countries. Thus, this study aims to clarify the factors related to turnover intention among rehabilitation professionals in Japan.

## Materials and methods

Recruitment

We conducted a cross-sectional survey in June 2017 using a web-based questionnaire. An Internet panel survey company, Macromill Co., Ltd. (Tokyo, Japan), was responsible for recruiting participants and collecting responses. Participants were recruited from the company’s registered panel members. The company randomly selected subjects who are/were PTs or OTs from a list and sent them an email inviting them to participate in the survey. Registration closed once the targeted sample size was reached. Participants completed the questionnaires through email and received a reward upon completion; the exact amount of the reward was kept confidential by the survey company.

Data collection

We used cross-sectional data from the web-based questionnaire that included information on participants’ backgrounds, intentions to leave their jobs, attitudes toward their jobs, and working environment, such as relationships with coworkers and abusive language and violence from patients and patients’ families. Thirty-three items were included in the questionnaire, which used a rating scale and multiple-choice questions. For example, participants were asked, “How is the atmosphere in your workplace?” Respondents then answered using a five-point Likert scale. Multiple-choice questions included “What is the reason for the bad atmosphere in the workplace?” The questionnaire is shown in Table 4 (Appendix).

Inclusion/exclusion criteria

Respondents working in government or educational institutions were excluded from the analysis based on responses to Q4 and Q5 in the Appendix. Thus, this study focused on PTs and OTs engaged in clinical work in hospitals, clinics, and nursing homes.

Statistical analysis

**Figure 1:**
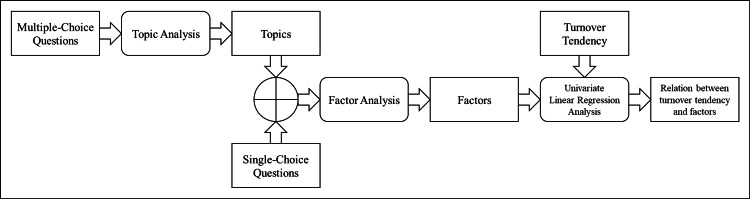
A simplified diagram of the analysis process in this study The rectangles shown in the figure represent numerical data, such as results based on questionnaire surveys and analyses. Rectangles with rounded corners indicate the analysis method used in each step. A cross on a circle indicates the integration of two sets of data.

The survey included a combination of multiple-choice and rating scale questions. One open-ended question was included; however, due to the low number of responses, that question was excluded from the analysis. Multiple-choice questions were analyzed using topic analysis developed by Blei et al. [25], performed in R 4.1.1 with the "topicmodels" package. Q25 was designated as the objective variable for the univariate linear regression. Q7, which required selecting one option from four, was split into PT and OT categories and used as input for the topic analysis. Single-choice questions (rating scale questions) were directly included in the factor analysis. A simplified diagram of the analysis process is shown in Figure 1.

The following is a summary of the analytical flow of this study. Through topic analysis, the relationship between documents, topics, and words was redefined as respondents, emotions, and survey responses, respectively, so that emotional topics were extracted, and their corresponding values were calculated for each respondent. Topics weakly associated with turnover intention were excluded, and the remaining topics were interpreted to assess their relevance. Factor analysis was conducted to summarize the extracted emotional topics and the scaled rating responses. Principal component analysis was performed to determine the appropriate number of factors, followed by factor analysis to extract and interpret the factor meanings.

Linear regression analyses were conducted, with the extracted factors as independent variables and turnover intention (Q25) as the dependent variable. Univariate linear regressions were performed to evaluate the relationships between each factor and turnover intention.

Ethics approval

This study was conducted with the approval of the Ethics Committee of Nara Medical University (authorization code: 1502). All participants provided informed consent (completed online) for data collection and storage. Written informed consent for study participation was obtained upon registration. The web-based questionnaire survey was conducted by an authorized survey company in adherence to personal information protection regulations. Anonymized data were obtained from the company after the completion of the survey.

## Results

A total of 515 people, 333 PTs and 182 OTs, responded. We selected people who were currently employed and excluded 34 potential participants who previously worked as rehabilitation professionals (currently unemployed) or who currently worked for the local government. A total of 481 participants were included: 312 (64.9%) PTs and 169 (35.1%) OTs; 247 (51.4%) males and 234 (48.6%) females; 94 (19.5%) in their 20s, 201 (41.8%) in their 30s, 131 (27.2%) in their 40s, and 55 (11.4%) in their 50s.

Tables 1, 2 show the responses to the single-choice (scaled) and multiple-choice questions, respectively, for items strongly related to turnover. For the multiple-choice questions, only the top five items are shown. The number of responses to the remaining questions is shown in Tables 5, 6 in the Appendix.

**Table 1:** Number of responses to questions (single choice) regarding items related to turnover Percentages are calculated as the ratio of the total number of physical therapists, occupational therapists, and combined totals. In Q25, which is about intention to quit, 6.4% of physical therapists and 10.1% of occupational therapists said, "I want to quit."

ID	Question statement	Answer	Physiotherapist	Occupational therapist	Total
Age	Ages	20-29	57(18.3%)	37(21.9%)	94(19.5%)
		30-39	129(41.3%)	72(42.6%)	201(41.8%)
		40-49	83(26.6%)	48(28.4%)	131(27.2%)
		50-59	43(13.8%)	12(7.1%)	55(11.4%)
Q2	At how many workplaces have you worked (including your current workplace)? Please limit your response to physiotherapist/occupational therapist jobs.	1	133(42.6%)	65(38.5%)	198(41.2%)
		2	88(28.2%)	43(25.4%)	131(27.2%)
		3	53(17.0%)	36(21.3%)	89(18.5%)
		4‒6	29(9.3%)	23(13.6%)	52(10.8%)
		7‒9	8(2.6%)	2(1.2%)	10(2.1%)
		10 or more	1(0.3%)	0(0.0%)	1(0.2%)
Q10S1	Please answer each of the following. Use the scores below: Are you satisfied with your current workload?	Satisfied	42(13.5%)	19(11.2%)	61(12.7%)
		Somewhat satisfied	180(57.7%)	96(56.8%)	276(57.4%)
		Slightly satisfied	65(20.8%)	40(23.7%)	105(21.8%)
		Dissatisfied	25(8.0%)	14(8.3%)	39(8.1%)
Q10S2	Please answer each of the following. Use the scores below: Are you satisfied with your current work hours?	Satisfied	65(20.8%)	33(19.5%)	98(20.4%)
		Somewhat satisfied	169(54.2%)	88(52.1%)	257(53.4%)
		Slightly satisfied	52(16.7%)	34(20.1%)	86(17.9%)
		Dissatisfied	26(8.3%)	14(8.3%)	40(8.3%)
Q10S3	Please answer each of the following. Use the scores below: Are you satisfied with your current salary/wages?	Satisfied	23(7.4%)	10(5.9%)	33(6.9%)
		Somewhat satisfied	98(31.4%)	49(29.0%)	147(30.6%)
		Slightly satisfied	109(34.9%)	75(44.4%)	184(38.3%)
		Dissatisfied	82(26.3%)	35(20.7%)	117(24.3%)
Q13	Do you currently have work-related stress?	None at all	3(1.0%)	1(0.6%)	4(0.8%)
		Almost none	67(21.5%)	32(18.9%)	99(20.6%)
		A small amount	141(45.2%)	70(41.4%)	211(43.9%)
		Quite a substantial amount	75(24.0%)	50(29.6%)	125(26.0%)
		Extremely high levels of stress	26(8.3%)	16(9.5%)	42(8.7%)
Q18	Do you feel fulfilled by the work in your current workplace?	I feel fulfilled	25(8.0%)	14(8.3%)	39(8.1%)
		I feel somewhat fulfilled	190(60.9%)	97(57.4%)	287(59.7%)
		I do not feel very fulfilled	87(27.9%)	51(30.2%)	138(28.7%)
		I do not feel fulfilled at all	10(3.2%)	7(4.1%)	17(3.5%)
Q21	How is the atmosphere at your current workplace?	Very good	26(8.3%)	13(7.7%)	39(8.1%)
		Fairly good	201(64.4%)	95(56.2%)	296(61.5%)
		Sometimes bad	71(22.8%)	49(29.0%)	120(24.9%)
		Quite bad	14(4.5%)	12(7.1%)	26(5.4%)
Q25	Have you ever thought you wanted to quit your current job? Please select the one answer that is most applicable to you.	I have never wanted to quit	62(19.9%)	19(11.2%)	81(16.8%)
		I do not want to quit, but I may change jobs if I found somewhere better than here	118(37.8%)	66(39.1%)	184(38.3%)
		I considered quitting, but then I stopped thinking about it	90(28.8%)	46(27.2%)	136(28.3%)
		I am planning on quitting and am looking for other employment	22(7.1%)	21(12.4%)	43(8.9%)
		I want to quit	20(6.4%)	17(10.1%)	37(7.7%)

**Table 2:** The top five responses to the possible reasons for turnover, Q26 and Q33 (multiple choice) Q26 asks why people are thinking of quitting, and Q33 asks why they left their previous job. Many people answered that they had not quit their previous job in Q33, but in both questions, the salary was often cited as the reason.

ID	Question	Rank	Answer	Response
Q26	What would be the trigger for you leaving your job? If you have any other answers, please write them in the free entry field. (May select multiple answers) Please answer this question even if you are not currently working.	1	I am not paid a salary commensurate with my work	234(48.6%)
		2	It is difficult to take holidays	139(28.9%)
		3	There is a lot of overtime	99(20.6%)
		4	Deterioration of my own health	88(18.3%)
		5	To have or raise children	66(13.7%)
Q33	Why did you quit your previous job? Please select up to five applicable answers from the following. (Multiple answers allowed)	1	I have never quit a job	176(36.6%)
		2	The income was not as good as I expected	71(14.8%)
		3	The workplace atmosphere was not good	71(14.8%)
		4	I was just really tired	53(11.0%)
		5	The work was too hard	49(10.2%)

The questionnaires contained 17 multiple-choice questions for a total of 240 potential choices. These 17 questions had an "other" option to allow respondents to write answers freely. However, this option was seldom used, and those who did use it did not give the same answer, so we omitted these responses. In other words, the topic analysis was performed assuming that the document could contain 240 types of words. We extracted two to fourteen topics, but the results of seven topics were easy to interpret, so we used these seven topics. These topics are as follows: “I want to do research activities”; “I don’t want to do research activities”; “Human relations in the workplace”; “Dissatisfaction with my previous workplace and satisfaction with my current workplace”; “Pleasant workplace, Career advancement intention”; “Work style: Early and late arrival”; “Working hours, salary, and other positive aspects of the work environment.” Table 7 in the Appendix shows the relations between topics and questionnaire items, which indicates the top 10 relations for each topic.

Since the correlation matrix of the topic values was singular, factor analysis could not be applied to the topic matrix, and we removed one topic (Topic 6), which had the weakest relationship with the target variable. Table 8 in the Appendix shows the results of univariate linear regression with the topic value of each respondent as the independent variable and intention to leave as the dependent variable.

Factor analysis was then conducted using six topics and 14 rating scale questions, extracting eight factors based on eigenvalues greater than 1. From the relationship of each loading amount, Factors 1 to 8 are, respectively, “satisfaction with work”; “motivation for current work”; “workplace size”; “working hours and salary”; “interpersonal relationships at work”; “motivation for research activities;” “dissatisfaction with previous workplace and satisfaction with current workplace”; and “years of experience.” Table 9 in the Appendix shows the results of factor analysis.

We executed univariate linear regressions for eight factors. Table 3 shows the results of the univariate linear regressions. Four factors had p-values less than 0.05, and four factors had p-values greater than 0.05. In other words, these factors were not directly related to the dependent variable. We interpreted the meaning of each factor with a p-value less than 0.05 as follows: Factor 1: People with low job satisfaction are more likely to quit. Factor 4: Even if the work environment is positive, they will quit because of the relationship between working hours and salary. Factor 5: Workplace relationships matter. Factor 6: Those who are highly motivated in research activities are more likely to quit.

**Table 3:** Results of univariate linear regressions between factors and turnover tendency *statistically significant; t-value: t statistics; p-value: probability The results of univariate linear regression with the factor value of each respondent as the independent variable and intention to leave as the dependent variable.

	Coefficient	t-value	p-value
Factor 1	0.20732	8.710	*0.000
Factor 2	0.03027	1.094	0.274
Factor 3	0.02075	0.796	0.426
Factor 4	0.05428	1.968	*0.050
Factor 5	0.19900	7.605	*0.000
Factor 6	-0.18299	-6.926	*0.000
Factor 7	0.04066	1.470	0.142
Factor 8	-0.03083	-1.259	0.209

## Discussion

Our study identified that four factors were associated with turnover intention among rehabilitation professionals in Japan: low job satisfaction, imbalance between working hours and salary, interpersonal relationships with coworkers, and high motivation for research activities. Our results mostly support previous studies about turnover among rehabilitation professionals and nurses in other countries.

Advantages of the topic analysis used in this study

In this study, we analyzed factors associated with turnover intention among PTs and OTs using topic analysis.

When conducting a questionnaire survey, scaled questions require respondents to choose from multiple options (scales) for a single keyword. Conversely, with multiple-choice questions, respondents can answer simply by checking the appropriate keywords; this reduces the time required to respond to a single keyword, thus allowing more keywords to be presented within the same response time. However, multiple-choice questions do not allow for scaled responses, which limits the analysis methods. Topic analysis is a powerful method for extracting latent themes by identifying word associations within text and is one of the analytical methods that address the previously mentioned limitations. For example, Zhao et al. [26] applied topic analysis to medical datasets, while Maeda and Jin [27] used this approach to reveal the characteristics of railway stations in urban areas. In our study, we adapted this method by replacing documents with respondents, topics with emotions, and words with questionnaire answers to explore emotional factors related to turnover intention. In our study, we adapted this method to analyze the results of questionnaire items related to turnover intentions by replacing documents with respondents, topics with emotions, and words with questionnaire answers. During the analysis process, we utilized multiple-choice questions to obtain categorical data and combined them with rating scale questions to quantify response intensity, allowing us to leverage the advantages of both.

Various confounding factors, such as work environment, years of experience, and salary level, may affect the intention to leave. While fully accounting for these confounders is challenging, our method involves factor analysis, which helps identify latent constructs by grouping similar response patterns. As a result, the confounding factors are incorporated within the identified factors, allowing for their indirect consideration in the analysis. Further analysis to clarify associations among the factors and the determinants for each factor could be beneficial for obtaining practically useful and specific knowledge to prevent turnover among rehabilitation professionals.

Turnover intention and low job satisfaction (factor 1)

Scanlan et al. [24] reported that job satisfaction influenced turnover intention among OTs. They administered questionnaires to 103 OTs working in mental health to explore the elements related to job satisfaction, turnover intention, and burnout. The results showed a relationship between lower job satisfaction and higher turnover intention. An association between nurse turnover and low job satisfaction has also been reported [19,20]. Liu et al. [19] surveyed 1706 nurses working at hospitals and revealed that turnover intention negatively correlated with job satisfaction and organizational support. Applebaum et al. [20] also reported a negative relationship between nurse turnover intention and job satisfaction. Our results support those findings. Regarding job satisfaction, a previous study [21] reported that freedom on the job and opportunity for skill development were positively associated with job satisfaction among PTs. Scanlan et al. [24] reported that higher job satisfaction was positively associated with reward and being highly valued by coworkers among OTs. High salaries, opportunities for skill development, and being valued by coworkers could improve job satisfaction, preventing turnover among rehabilitation professionals.

Turnover intention and imbalance between working hours and salary (factor 4)

A previous study for rehabilitation professionals reported an association between work retention and salary. Okerlund et al. [21] surveyed 590 PTs in the United States to determine the factors contributing to recruitment and retention. They identified pay and benefits as factors of retention. The same relationship between salary and turnover has been reported in the research on nurses [19,20]. Salary might be a common factor in health professional turnover. Further analysis, including the factor’s elements (e.g., work content, responsibilities, position, and overtime work), might generate more pragmatic knowledge on this topic.

Turnover intention and interpersonal relationships with coworkers (factor 5)

A previous study [23] clarified the association between turnover intention and interpersonal relationships with coworkers among OTs. This study targeted 257 OTs, and the results showed that turnover intention was positively associated with occupational stress, including interpersonal conflict. This association has also been reported in research on nurses [16]. In addition to salary, interpersonal relationships might contribute to health professional turnover.

Turnover intention and high motivation in research activities (factor 6)

A positive relationship between turnover intention and motivation for research activities has not been reported for rehabilitation professionals. A previous study on nurses showed that turnover intention is negatively associated with their motivation to improve their professional capabilities [18], which is the opposite of our result. In our study, research activities included the presentation of case reports in the workplace. Thus, this factor might widely indicate a motivation to improve their capacities, such as improving their specialty, admission to graduate school, pursuing academic careers, and so on, which could influence turnover intention in various ways.

Limitations

Potential selection biases in this study may limit the generalizability of the results. One such bias is the age distribution of participants, with the largest age group being 30-39. Additionally, this study only included participants who had access to and could respond using the Internet. The data were collected solely from respondents who were registered with an Internet panel company, which could introduce bias. Nevertheless, panel-based surveys have been widely used in recent years [28-30]. The participants received a small cash reward for completing the survey, which might have influenced the representativeness of the sample. However, as the research firm created the panel based on a large enrollment, the risk of bias is expected to be minimized. The other potential bias concerns participants' occupations. This study included 333 PTs and 182 OTs but no speech-language-hearing therapists. The number of PTs was roughly twice that of OTs, which reflects their distribution in Japan.

## Conclusions

This study aimed to identify factors related to turnover intention among PTs and OTs engaged in clinical practice in hospitals, clinics, and nursing homes. Grounded in theories of job satisfaction and organizational behavior, it examined workplace environment relationships with colleagues and experiences of verbal abuse or violence from patients and their families. Topic analysis was conducted on multiple-choice questions to categorize respondents' emotions, extracting seven topics, while factor analysis of rating scale responses identified eight key factors through principal component analysis. Univariate linear regression was employed to assess associations between these factors and turnover intention. Our study revealed that four factors were significantly associated with turnover intention: low job satisfaction, an imbalance between working hours and salary, interpersonal relationships in the workplace, and high motivation for research activities. These results suggest that both intrinsic and extrinsic work factors influence retention. Notably, those with lower job satisfaction and poor workplace relationships exhibited a stronger intention to leave. These findings suggest that improving workplace relationships and increasing job satisfaction may help reduce turnover among rehabilitation professionals.

## Appendices

**Table 4:** Questionnaire items The table shows the questionnaire items administered in this study. Q4 and Q5 were used to select respondents. Q25 was used as the final dependent variable, which was intention to leave. Questions that allowed for multiple answers were used as input for topic analysis. Q7 was considered a yes/no question for physical therapists and occupational therapists and was used as input for topic analysis. Q34 is an open-ended question. Because only a small number of people responded, it was not used in the analysis. The remaining single-choice questions were used as input for factor analysis. This questionnaire was prepared by M. A., T. A., and A. K.

ID	Question statement	Choices
Q1	Please tell us how many years you worked as a physiotherapist/occupational therapist (how many years’ experience). Please state the total number of years, including cases where you retired and then started working again. If you have worked at several workplaces, please state the total number of years.	0‒3 years / 3‒6 years / 6‒9 years / 9‒15 years / 15‒20 years / 20‒25 years / 25‒30 years / Longer than 30 years
Q2	At how many workplaces have you worked (including your current workplace)? Please limit your response to physiotherapist/occupational therapist jobs.	1 / 2 / 3 / 4‒6 / 7‒9 / 10 or more
Q3	Please tell us your highest level of education.	Graduated from a vocational school / Graduated from a junior college / Graduated from a 4-year university / Completed post-graduate studies (Master’s degree) / Completed post-graduate studies (Doctorate) / Other ( )
Q4	Please choose the applicable answer.	Currently working / Currently on leave
Q5	Please tell us about your current place of employment. If you work at several workplaces, please answer regarding your main workplace. (Also applies to the following questions.)	Hospital with 500 or more beds / Hospital with 200‒499 beds / Hospital with fewer than 200 beds / Clinic with beds / Clinic without beds (medical office) / Health and welfare facility for older individuals / Long-term care medical facility / Home-visit nursing station / Local government (public servant, etc.) / Educational organization (university or vocational school, etc.) / Other facility (________)
Q6	Please tell us how many people work at your current workplace as physiotherapists and occupational therapists. Please include yourself in the number of people.	
Q6S1	Physiotherapist	100 or more people / 30‒99 people / 10‒29 people / 5‒9 people / 2‒4 people / 0‒1 person
Q6S2	Occupational therapist	100 or more people / 30‒99 people / 10‒29 people / 5‒9 people / 2‒4 people / 0‒1 person
Q7	Is there a sufficient number of physiotherapists and occupational therapists employed at your current workplace?	Sufficient number of both / Sufficient number of physiotherapists / Sufficient number of occupational therapists / Insufficient number of both
Q8	Please indicate your current working arrangements. (Multiple answers allowed)	Day shifts / There are night shifts / There are early departures / There are late departures / I have weekends off / Irregular holidays
Q9	Please indicate your position at work.	Staff member / Chief / Workplace manager (chief engineer, department manager, clinical department manager, etc.) / Manager / Other (please state specifically) (____)
Q10	Please answer each of the following. Use the scores below:	
Q10S1	Are you satisfied with your current workload?	Satisfied / Somewhat satisfied / Slightly satisfied / Dissatisfied
Q10S2	Are you satisfied with your current work hours?	Satisfied / Somewhat satisfied / Slightly satisfied / Dissatisfied
Q10S3	Are you satisfied with your current salary/wages?	Satisfied / Somewhat satisfied / Slightly satisfied / Dissatisfied
Q11	What is the total number of hours you work each week, including overtime?	Less than 30 hours / More than 30 but less than 40 hours / More than 40 but less than 50 hours / More than 50 but less than 60 hours / 60 hours or more
Q12	What do you feel is the cause of your long working hours? (Multiple answers allowed)	Insufficient staff for the normal workload / Need to spend time improving my skills or working on my career in addition to my normal work / Need to spend time educating or instructing junior staff in addition to my normal work / Lack the ability to process my own work / Lack the ability to manage work / Events and meetings are held outside working hours / Seasonal differences in the workload / Workplace atmosphere (my colleagues stay back at work, so it is difficult for me to go home) / Other (___)
Q13	Do you currently have work-related stress?	None at all / Almost none / A small amount / Quite a substantial amount / Extremely high levels of stress
Q14	Select the applicable answer to describe your mental and physical health. Please select only one that is the most applicable to you.	I am healthy / Fatigue does not last until the following day / I always feel tired / I am aware of symptoms, such as sleep disorders / I am currently seeing a doctor at a hospital for stress / I am currently on leave
Q15	What kind of stress do you feel at work? (May select multiple answers) People who are currently not working should answer about your most recent place of work.	Heavy workload / Long working hours / Heavy responsibility / Bad human relations in the workplace / Difficult to communicate with patients / Bad workplace environment / No one to talk to / Lack of staff / Unstable employment / Anxiety about the future / The work does not suit my aptitude / I cannot keep up with the latest rehabilitation medicine / Other (_____)
Q16	Do you do anything to maintain your work and health? (Multiple answers allowed)	I take holidays / I spend time with my family / I play sports regularly / I have a hobby / I find purpose in my work / Other (____) / I don’t do anything
Q17	Why did you choose your current place of work? Please select up to five applicable answers from the following. (Multiple answers allowed)	Because the salary (base salary) is good / Because the bonuses are good / Because I received a scholarship / Because the housing allowance and overtime allowance are good / Because there are apartments or dormitories, and I received preferential treatment for moving in / Because I can take holidays as per the legal number of days / Because I can be employed as per my preferred working arrangements / Because there is little overtime / Because there are no nighttime consultations / Because the hospital (facility) has childcare / Because there is a system that makes it easy to take maternity leave, childcare leave, and caregiver leave / Because the size of the hospital (facility) was exactly what I wanted / Because the workplace atmosphere was good when I visited during job hunting / Because I have visited before, and the staff were very helpful / Because it is a clean and tidy hospital (facility) / Because the surrounding environment is good / Because the commute is good /Because the shopping and restaurant district around the closest railway station to my workplace is convenient / Because the hospital (facility) has a good reputation / Because it is a famous hospital (facility), often reported on television, etc. / Because the hospital (facility) is working on advanced medical care / Because it is a specialist hospital (facility) in my field of interest / Because it has a thorough education system for new staff / Because the education system within the hospital (facility) is thorough / Because there is support for participation in external training (training, seminars, and academic societies outside the hospital/facility) / Because it is the hospital (facility) where I trained / Because I have acquaintances working there / Because I intend to give back because my family was looked after at the hospital/facility / Because I have a family relationship with the manager / Because I was enthusiastically recruited / Because I was strongly recommended by the school (university) from which I graduated / Because I wanted to live in this city / Because there is a graduate school / Because the environment enables me to research / Other (____)
Q18	Do you feel fulfilled by the work in your current workplace?	I feel fulfilled / I feel somewhat fulfilled / I do not feel very fulfilled / I do not feel fulfilled at all
Q19	This question is for people who answered “I feel fulfilled” or “I feel somewhat fulfilled” in the previous question. How do you achieve a sense of fulfillment? Please select all applicable answers from the following. (Multiple answers allowed)	I am able to help patients and their families / I am trusted by patients and their families /I can teach my knowledge and skills to new staff and students / I can improve my own knowledge and ability / I can learn a great deal from senior staff / I can receive proper instructions and support from my boss / I can freely participate in lectures and training / I can conduct research / I can participate in planning and preparation / I feel my enthusiasm for and efforts at work are properly evaluated / I can advance to post-graduate studies while working / I can make friends / I can expand my horizons / I can live with financial stability / I have time for my hobbies / I can gain experience in rehabilitation for various diseases / There is a full range of treatment and evaluation equipment / Study groups within the hospital are fully established / I am able to increase my expertise / There is little overtime / Other (____)
Q20	Objectively speaking, what are the strengths of your current workplace? Please select all applicable answers from the following. (Multiple answers allowed)	Good quality of medical care / Active research activities / Interaction with other professions (doctors, pharmacists, nurses, etc.) / No conflict between different occupations / Convenient transportation / Good salary / Childcare center within or close to the hospital / Able to take paid leave as per the legal number of days / Few occasions of working on holidays / No or little overtime / Able to talk with my boss or manager about anything / Human relationships in the workplace are good / There is a break room / The changing rooms are bright and clean / The entire hospital is bright and clean / Other (______) / Nothing in particular
Q21	How is the atmosphere at your current workplace?	Very good / Fairly good / Sometimes bad / Quite bad
Q22	This question is for people who answered that the workplace atmosphere is “sometimes bad” or “quite bad” in the previous question. What are the reasons for your answer? Please select all applicable answers from the following. (Multiple answers allowed)	There is physical and verbal abuse from patients and patient’s families / I do not get on with my colleagues / There is no sense of comradeship among colleagues / I hear colleagues bad-mouthing and insulting other colleagues when they are not there / Important information is not communicated / I am ignored even if I greet people / I am left out of things / I am not taught the correct knowledge or method / The work atmosphere discourages me from asking about things I do not understand / My boss tends to talk like s/he is issuing orders / My boss rebukes me in front of my colleagues and the patients / I have a boss who only sees the negative side / I have a boss who picks up on shortcomings I am concerned / I am always rebuked for things that other people have done / I may be reluctantly underrated / No one listens to my opinion on operations and improvements / There is a doctor with an arrogant or overbearing attitude / There are doctors, nurses, staff, or officers who use offensive language and behavior / Cases of harassment (sexual harassment and power harassment) are becoming public / Management tells me to quit my job / Other (______)
Q23	Do you have any of the following problems at your current workplace? If you answered yes, please select all applicable answers from the following. (Multiple answers allowed)	There is a lack of consideration for patients’ human rights / There are many accidents and minor incidents / There are many complaints from patients and their families / Management prioritizes pursuit of profit / Frugality is too thorough / Large disparity in wages between different occupations / Many people take sick leave / High employee turnover / Working without pay before and after normal working hours is normalized / Not enough time for meals and breaks / There is harassment from management and staff / New people are bullied / Other ( ) / Nothing in particular
Q24	What is your specific impression of your current workplace? If you have other impressions, please state them in the free entry field. (May select multiple answers)	It is a comfortable workplace / It is easy to take breaks / Human relations are good / Staff helps each other / There is substantial welfare / There is no future here / Quotas and responsibilities are too heavy / The work is boring / The work does not lead to skill improvement / Other (_____)
Q25	Have you ever thought you wanted to quit your current job? Please select the one answer that is most applicable to you.	I have never wanted to quit / I do not want to quit, but I may change jobs if I found somewhere better than here / I considered quitting, but then I stopped thinking about it / I am planning on quitting and am looking for other employment / I want to quit
Q26	What would be the trigger for you leaving your job? If you have any other answers, please write them in the free entry field. (May select multiple answers) Please answer this question even if you are not currently working.	I am not paid a salary commensurate with my work / There is a lot of overtime / It is difficult to take holidays / To care for my family / To get married / To have or raise children / To undertake research or further study / Deterioration of my own health / Because of harassment (sexual harassment or power harassment) / Long commute / Other (______)
Q27	Is there anything you want from your current workplace? Please select all applicable answers from the following. (Multiple answers allowed)	I would like working conditions to improve to prevent burnout / I would like a guarantee that time will be made available to develop my career, such as progressing to post-graduate studies / Fully-equipped changing rooms and break rooms / I would like to strengthen risk management measures in the workplace, including accident prevention / I would like opinions on the workplace to be properly heard, such as when handling complaints from patients / I would like all staff to be thoroughly informed about medical ethics and patient human rights / I would like a fair evaluation of ability / I would like proper responses to sexual harassment / I would like measures to be established to prevent power harassment / I would like treatment time secured for each patient / I would like an environment that enables research / I would like an environment that allows participation in workshops and academic societies / Increased salary / Enhanced childcare / Encouragement to take annual leave / I would like an environment where it is easy to take maternity leave, childcare leave, and caregiver leave / Other (____)
Q28	What do you want from management or your boss? Please select all applicable answers from the following. (Multiple answers allowed) Please answer this question even if you are not currently working.	I would like information to be promptly provided to all staff (I do not want certain people singled out to not be informed about the information) / I would like management to not bad mouth people around me / I do not want to be spoken to in a tone that makes me feel cornered / I do not want to be patronized / I do not want to be looked down on / If I greet you, I would like to be greeted in return / I do not want to be asked about my private life / I do not want my ability to be evaluated based on my educational background or graduate school / I would also like you to see the positive side / I would like my enthusiasm and efforts at work to also be evaluated / I would like the evaluation using a demerit method to stop / I would like my opinion to be heard / I do not want unilateral decisions to be made / I do not want to be made to feel stupid when being taught (I would like to be treated equally even if I have different skills and knowledge) / I would like management to be kind and listen to me / I would like to be able to take annual leave / I would like to be able to take maternity leave, childcare leave, and caregiver leave / Other (____)
Q29	How do you feel about physiotherapists and occupational therapists conducting research? Note: “Research activities” here refers to activities undertaken with the aim of presenting study results and submitting papers related to rehabilitation-related issues, such as physical therapy and occupational therapy at academic societies and study groups, in-hospital and in-facility presentations.	I definitely think it is necessary / I think it is good to conduct research / It is not necessary / Absolutely not necessary
Q30	Would you like to undertake research activities? Note: “Research activities” here refers to activities undertaken with the aim of presenting study results and submitting papers related to rehabilitation-related issues, such as physical therapy and occupational therapy at academic societies and study groups, in-hospital and in-facility presentations.	I definitely want to / I would like to if I had the opportunity / I would prefer not to / I definitely do not want to
Q31	This question is for people who answered “I definitely want to” or “I would like to if I had the opportunity” in the previous question. What are the reasons for this answer? Please select all applicable answers from the following. (Multiple answers allowed)	Because I want to develop my specialization / Because I want to know more about rehabilitation medicine / Because it is necessary to advance my career / Because I will be highly regarded by my peers / Because I want to do something useful for my patients / Because I want to work overseas / Because it will lead to improved quality of rehabilitation / Because it will lead to improved social standing of physiotherapists and occupational therapists / Because I want to become a university faculty member / Other (____)
Q32	This question is for people who answered “I would prefer not to” or “I definitely do not want to” in the previous question. What are the reasons for this answer? Please select all applicable answers from the following. (Multiple answers allowed)	It would be a source of stress for me / I have unpleasant memories of being forced to conduct research in the past / There are not many studies that are useful for society / Research is far removed from actual rehabilitation / Practicing rehabilitation is more rewarding for me / I have no intention of aiming for a high degree of education / I will lose my free time / I do not consider researchers to be distinguished / I have been bullied by a university teacher / I have no interest in research / Research does not lead to clinical practice / Other (___)
Q33	Why did you quit your previous job? Please select up to five applicable answers from the following. (Multiple answers allowed)	The income was not as good as I expected / The scholarship refund exemption period had expired / No allowances were paid for overtime / The work was too hard / I could not take adequate holidays / There were too many meetings and study sessions outside work hours / I had to go to work before my rostered start time / There was a lot of overtime / The work hours were not flexible for childcare or long-term care / Could not take adequate paid leave / I often had to go to work on my days off / The workplace atmosphere was not good / I was subjected to power harassment and/or sexual harassment / I was harassed by a colleague / Interactions with colleagues outside my own profession were bad / My good friend and/or colleague quit / The commute was inconvenient / The new employee training system was inadequate / I was suddenly forced to do work that required experience / My instructor did not instruct me correctly / I wanted to experience rehabilitation work in other fields (acute phase, convalescence phase, maintenance phase) / There is no education system in the hospital / There is no support for attending training or seminars for career development / No consideration was given to me for balancing work and study when I progressed to post-graduate studies / I progressed to higher education, so I wanted to concentrate on my studies / I got married / I managed to save enough money to do the things I want to do while I’m still young / I became busy with my family, such as childcare and long-term care / I was just really tired / My own health had deteriorated / I wanted to work outside my own profession / I heard that the hospital management was in danger /I received a bad evaluation / I made a mistake, which made it hard for me to stay there / A patient complained about me / I was subjected to violence by a patient (including harassment), and I became scared / Other (_________) / I have never quit a job
Q34	What are your impressions or image of your work? (Including treating patients, educating students, research activities, etc.) People who are on leave (in between jobs) should also answer this question.	

**Table 5:** The top five responses to the multiple-choice questions not included in Table 2 Q17 had two choices with the same number of responses for the fifth place, so six choices are listed.

ID	Question Statement	Rank	Answer	Response
Q8	Please indicate your current working arrangements. (Multiple answers allowed)	1	Day shifts	457(95.0%)
		2	Irregular holidays	125(26.0%)
		3	I have weekends off	83(17.3%)
		4	There are early departures	32(6.7%)
		5	There are late departures	21(4.4%)
Q12	What do you feel is the cause of your long working hours? (Multiple answers allowed)	1	Insufficient staff for the normal workload	184(38.3%)
		2	Events and meetings are held outside working hours	168(34.9%)
		3	Need to spend time improving my skills or working on my career in addition to my normal work	134(27.9%)
		4	Seasonal differences in the workload	125(26.0%)
		5	Need to spend time educating or instructing junior staff in addition to my normal work	119(24.7%)
Q15	What kind of stress do you feel at work? (May select multiple answers) People who are currently not working should answer about your most recent place of work.	1	Anxiety about the future	163(33.9%)
		2	Heavy workload	149(31.0%)
		3	Bad human relations in the workplace	134(27.9%)
		4	Heavy responsibility	113(23.5%)
		5	Lack of staff	113(23.5%)
Q16	Do you do anything to maintain your work and health? (Multiple answers allowed)	1	I take holidays	234(48.6%)
		2	I have a hobby	193(40.1%)
		3	I spend time with my family	172(35.8%)
		4	I play sports regularly	152(31.6%)
		5	I find purpose in my work	84(17.5%)
Q17	Why did you choose your current place of work? Please select up to five applicable answers from the following. (Multiple answers allowed)	1	Because the commute is good	121(25.2%)
		2	Because I can take holidays as per the legal number of days	112(23.3%)
		3	Because it is a specialist hospital (facility) in my field of interest	100(20.8%)
		4	Because I can be employed as per my preferred working arrangements	86(17.9%)
		5	Because the salary (base salary) is good	80(16.6%)
		5	Because the workplace atmosphere was good when I visited during job hunting	80(16.6%)
Q19	This question is for people who answered “I feel fulfilled” or “I feel somewhat fulfilled” in the previous question. How do you achieve a sense of fulfillment? Please select all applicable answers from the following. (Multiple answers allowed)	1	I am able to help patients and their families	195(40.5%)
		2	I am trusted by patients and their families	156(32.4%)
		3	I can improve my own knowledge and ability	91(18.9%)
		4	I can live with financial stability	89(18.5%)
		5	I can gain experience in rehabilitation for various diseases	71(14.8%)
Q20	Objectively speaking, what are the strengths of your current workplace? Please select all applicable answers from the following. (Multiple answers allowed)	1	Few occasions of working on holidays	156(32.4%)
		2	Able to take paid leave as per the legal number of days	154(32.0%)
		3	Human relationships in the workplace are good	142(29.5%)
		4	Interaction with other professions (doctors, pharmacists, nurses, etc.)	139(28.9%)
		5	No or little overtime	135(28.1%)
Q22	This question is for people who answered that the workplace atmosphere is “sometimes bad” or “quite bad” in the previous question. What are the reasons for your answer? Please select all applicable answers from the following. (Multiple answers allowed)	1	There is no sense of comradeship among colleagues	61(12.7%)
		2	Important information is not communicated	52(10.8%)
		3	I hear colleagues bad-mouthing and insulting other colleagues when they are not there	51(10.6%)
		4	I do not get on with my colleagues	43(8.9%)
		5	I have a boss who only sees the negative side	40(8.3%)
Q23	Do you have any of the following problems at your current workplace? If you answered yes, please select all applicable answers from the following. (Multiple answers allowed)	1	Nothing in particular	127(26.4%)
		2	Management prioritizes the pursuit of profit	111(23.1%)
		3	High employee turnover	95(19.8%)
		4	There is a lack of consideration for patients’ human rights	93(19.3%)
		5	Working without pay before and after normal working hours is normalized	91(18.9%)
Q24	What is your specific impression of your current workplace? If you have other impressions, please state them in the free entry field. (May select multiple answers)	1	It is easy to take breaks	203(42.2%)
		2	It is a comfortable workplace	200(41.6%)
		3	Human relations are good	156(32.4%)
		4	There is no future here	133(27.7%)
		5	Staff help each other	123(25.6%)
Q27	Is there anything you want from your current workplace? Please select all applicable answers from the following. (Multiple answers allowed)	1	Increased salary	306(63.6%)
		2	Encouragement to take annual leave	138(28.7%)
		3	I would like a fair evaluation of ability	121(25.2%)
		4	I would like working conditions to improve to prevent burnout	111(23.1%)
		5	I would like all staff to be thoroughly informed about medical ethics and patient human rights	95(19.8%)
Q28	What do you want from management or your boss? Please select all applicable answers from the following. (Multiple answers allowed) *Please answer this question even if you are not currently working.	1	I would like information to be promptly provided to all staff (I do not want certain people singled out to not be informed about the information)	185(38.5%)
		2	I do not want unilateral decisions to be made	111(23.1%)
		3	I do not want to be patronized	83(17.3%)
		4	I would like management to not bad mouth people around me	82(17.0%)
		5	I do not want to be spoken to in a tone that makes me feel cornered	79(16.4%)
Q31	This question is for people who answered “I definitely want to” or “I would like to if I had the opportunity” in the previous question. What are the reasons for this answer? Please select all applicable answers from the following. (Multiple answers allowed)	1	Because I want to develop my specialization	182(37.8%)
		2	Because it will lead to improved quality of rehabilitation	136(28.3%)
		3	Because I want to do something useful for my patients	123(25.6%)
		4	Because it is necessary to advance my career	110(22.9%)
		5	Because it will lead to improved social standing of physiotherapists and occupational therapists	85(17.7%)
Q32	This question is for people who answered “I would prefer not to” or “I definitely do not want to” in the previous question. What are the reasons for this answer? Please select all applicable answers from the following. (Multiple answers allowed)	1	It would be a source of stress for me	151(31.4%)
		2	I will lose my free time	94(19.5%)
		3	Practicing rehabilitation is more rewarding for me	91(18.9%)
		4	I have no interest in research	85(17.7%)
		5	I have no intention of aiming for a high degree of education	54(11.2%)

**Table 6:** Number of responses to questions (single choice) other than regarding items related to turnover Percentages are calculated as the ratio of the total number of physical therapists, occupational therapists, and combined totals.

ID	Question statement	Answer	Physiotherapist	Occupational therapist	Total
Q1	At how many workplaces have you worked (including your current workplace)? Please limit your response to physiotherapist/occupational therapist jobs.	0‒3 years	15(4.8%)	11(6.5%)	26(5.4%)
		3‒6 years	43(13.8%)	28(16.6%)	71(14.8%)
		6‒9 years	52(16.7%)	33(19.5%)	85(17.7%)
		9‒15 years	102(32.7%)	60(35.5%)	162(33.7%)
		15‒20 years	38(12.2%)	17(10.1%)	55(11.4%)
		20‒25 years	28(9.0%)	12(7.1%)	40(8.3%)
		25‒30 years	21(6.7%)	6(3.6%)	27(5.6%)
		Longer than 30 years	13(4.2%)	2(1.2%)	15(3.1%)
Q3	Please tell us your highest level of education.	Graduated from a vocational school	188(60.3%)	91(53.8%)	279(58.0%)
		Graduated from a junior college	14(4.5%)	13(7.7%)	27(5.6%)
		Graduated from a 4-year university	93(29.8%)	59(34.9%)	152(31.6%)
		Completed post-graduate studies (Master’s degree)	15(4.8%)	6(3.6%)	21(4.4%)
		Completed post-graduate studies (Doctorate)	1(0.3%)	(0.0%)	1(0.2%)
		Other ( )	1(0.3%)	(0.0%)	1(0.2%)
Q6S1	Please tell us how many people work at your current workplace as physiotherapists and occupational therapists. Please include yourself in the number of people: physiotherapists	100 or more people	6(1.9%)	(0.0%)	6(1.2%)
		30‒99 people	59(18.9%)	28(16.6%)	87(18.1%)
		10‒29 people	76(24.4%)	41(24.3%)	117(24.3%)
		5‒9 people	59(18.9%)	22(13.0%)	81(16.8%)
		2‒4 people	81(26.0%)	37(21.9%)	118(24.5%)
		0‒1 person	31(9.9%)	41(24.3%)	72(15.0%)
Q6S2	Please tell us how many people work at your current workplace as physiotherapists and occupational therapists. Please include yourself in the number of people: occupational therapists	100 or more people	2(0.6%)	(0.0%)	2(0.4%)
		30‒99 people	27(8.7%)	14(8.3%)	41(8.5%)
		10‒29 people	65(20.8%)	44(26.0%)	109(22.7%)
		5‒9 people	45(14.4%)	38(22.5%)	83(17.3%)
		2‒4 people	69(22.1%)	58(34.3%)	127(26.4%)
		0‒1 person	104(33.3%)	15(8.9%)	119(24.7%)
Q7	Is there a sufficient number of physiotherapists and occupational therapists employed at your current workplace?	Sufficient number of both	130(41.7%)	68(40.2%)	198(41.2%)
		Sufficient number of physiotherapists	25(8.0%)	10(5.9%)	35(7.3%)
		Sufficient number of occupational therapists	61(19.6%)	37(21.9%)	98(20.4%)
		Insufficient number of both	96(30.8%)	54(32.0%)	150(31.2%)
Q9	Please indicate your position at work.	Staff member	209(67.0%)	133(78.7%)	342(71.1%)
		Chief	50(16.0%)	29(17.2%)	79(16.4%)
		Workplace manager (chief engineer, department manager, clinical department manager, etc.)	35(11.2%)	6(3.6%)	41(8.5%)
		Manager	11(3.5%)	0(0.0%)	11(2.3%)
		Other (please state specifically) (____)	7(2.2%)	1(0.6%)	8(1.7%)
Q11	What is the total number of hours you work each week, including overtime?	Less than 30 hours	40(12.8%)	26(15.4%)	66(13.7%)
		More than 30 but less than 40 hours	63(20.2%)	32(18.9%)	95(19.8%)
		More than 40 but less than 50 hours	150(48.1%)	83(49.1%)	233(48.4%)
		More than 50 but less than 60 hours	48(15.4%)	24(14.2%)	72(15.0%)
		60 hours or more	11(3.5%)	4(2.4%)	15(3.1%)
Q14	Select the applicable answer to describe your mental and physical health. Please select only one that is the most applicable to you.	I am healthy	65(20.8%)	28(16.6%)	93(19.3%)
		Fatigue does not last until the following day	104(33.3%)	57(33.7%)	161(33.5%)
		I always feel tired	123(39.4%)	65(38.5%)	188(39.1%)
		I am aware of symptoms, such as sleep disorders	14(4.5%)	11(6.5%)	25(5.2%)
		I am currently seeing a doctor at a hospital for stress	6(1.9%)	7(4.1%)	13(2.7%)
		I am currently on leave	0(0.0%)	1(0.6%)	1(0.2%)
Q29	How do you feel about physiotherapists and occupational therapists conducting research? Note: “Research activities” here refers to activities undertaken with the aim of presenting study results and submitting papers related to rehabilitation-related issues, such as physical therapy and occupational therapy at academic societies and study groups, in-hospital and in-facility presentations.	I definitely think it is necessary	74(23.7%)	33(19.5%)	107(22.2%)
		I think it is good to conduct research	200(64.1%)	124(73.4%)	324(67.4%)
		It is not necessary	28(9.0%)	10(5.9%)	38(7.9%)
		Absolutely not necessary	10(3.2%)	2(1.2%)	12(2.5%)
Q30	Would you like to undertake research activities? Note: “Research activities” here refers to activities undertaken with the aim of presenting study results and submitting papers related to rehabilitation-related issues, such as physical therapy and occupational therapy at academic societies and study groups, in-hospital and in-facility presentations.	I definitely want to	33(10.6%)	16(9.5%)	49(10.2%)
		I would like to if I had the opportunity	137(43.9%)	71(42.0%)	208(43.2%)
		I would prefer not to	95(30.4%)	67(39.6%)	162(33.7%)
		I definitely do not want to	47(15.1%)	15(8.9%)	62(12.9%)

**Table 7:** Relations between topics and questionnaire items The top 10 questionnaire items strongly related to each topic (topic numbers 1-7) are shown. The percentage indicates the strength of each item, and the sum of all values for each topic (including the items after the 10th place shown in the table) is 1. In this study, each topic was given a name based on the questionnaire items that were strong for the topic.

Topic No.	1	2	3	4	5	6	7
Topic Name	I want to do research activities	I don’t want to do research activities	Human relations in the workplace	Dissatisfaction with my previous workplace and satisfaction with my current workplace	Pleasant workplace, Career advancement intention	Work style: Early and late arrival	Working hours, salary, and other positive aspects of the work environment
1	5.91%	4.34%	7.28%	5.95%	4.01%	49.03%	3.36%
	This question is for people who answered “I definitely want to” or “I would like to if I had the opportunity” in the previous question. Because I want to develop my specialization	This question is for people who answered “I would prefer not to” or “I definitely do not want to” in the previous question. I have no interest in research	What kind of stress do you feel at work? Bad human relations in the workplace	What is your specific impression of your current workplace? It is a comfortable workplace	What is your specific impression of your current workplace? It is a comfortable workplace	Please indicate your current working arrangements. There are early departures	Please indicate your current working arrangements. Day shifts
2	4.94%	4.06%	6.70%	4.73%	3.93%	39.60%	3.16%
	This question is for people who answered “I definitely want to” or “I would like to if I had the opportunity” in the previous question. Because it will lead to improved quality of rehabilitation	What is your specific impression of your current workplace? It is a comfortable workplace	This question is for people who answered that the workplace atmosphere is “sometimes bad” or “quite bad” in the previous question. There is no sense of comradeship among colleagues	Why did you quit your previous job? Please select up to five applicable answers from the following. The income was not as good as I expected	This question is for people who answered “I feel fulfilled” or “I feel somewhat fulfilled” in the previous question. I am able to help patients and their families	Please indicate your current working arrangements. There are late departures	Is there anything you want from your current workplace? Increased salary
3	4.55%	4.06%	5.82%	4.43%	3.69%	11.32%	2.53%
	This question is for people who answered “I definitely want to” or “I would like to if I had the opportunity” in the previous question. Because I want to do something useful for my patients	This question is for people who answered “I would prefer not to” or “I definitely do not want to” in the previous question. I will lose my free time	This question is for people who answered that the workplace atmosphere is “sometimes bad” or “quite bad” in the previous question. I have a boss who only sees the negative side	This question is for people who answered “I definitely want to” or “I would like to if I had the opportunity” in the previous question. Because I want to develop my specialization	This question is for people who answered “I feel fulfilled” or “I feel somewhat fulfilled” in the previous question. I am trusted by patients and their families	What kind of stress do you feel at work? ： Bad workplace environment	Is there a sufficient number of physiotherapists and occupational therapists employed at your current workplace? Insufficient number of occupational therapists
4	3.29%	3.99%	5.53%	4.27%	3.61%	0.00%	2.29%
	This question is for people who answered “I definitely want to” or “I would like to if I had the opportunity” in the previous question. Because it will lead to improved social standing of physiotherapists and occupational therapists	This question is for people who answered “I would prefer not to” or “I definitely do not want to” in the previous question. It would be a source of stress for me	This question is for people who answered that the workplace atmosphere is “sometimes bad” or “quite bad” in the previous question. Important information is not communicated	This question is for people who answered “I feel fulfilled” or “I feel somewhat fulfilled” in the previous question. I am able to help patients and their families	This question is for people who answered “I definitely want to” or “I would like to if I had the opportunity” in the previous question. Because I want to develop my specialization	What is your specific impression of your current workplace? It is a comfortable workplace	What would be the trigger for you leaving your job? I am not paid a salary commensurate with my work
5	3.19%	3.85%	5.53%	4.12%	3.54%	0.00%	1.99%
	This question is for people who answered “I definitely want to” or “I would like to if I had the opportunity” in the previous question. Because it is necessary to advance my career	Objectively speaking, what are the strengths of your current workplace? Able to take paid leave as per the legal number of days	This question is for people who answered that the workplace atmosphere is “sometimes bad” or “quite bad” in the previous question. I hear colleagues bad-mouthing and insulting other colleagues when they are not there	Why did you quit your previous job? Please select up to five applicable answers from the following No allowances were paid for overtime	What is your specific impression of your current workplace? Human relations are good	This question is for people who answered “I feel fulfilled” or “I feel somewhat fulfilled” in the previous question. I am able to help patients and their families	What do you feel is the cause of your long working hours? Insufficient staff for the normal workload
6	3.00%	3.72%	5.39%	3.97%	2.99%	0.00%	1.91%
	What is your specific impression of your current workplace? The work does not lead to skill improvement	Objectively speaking, what are the strengths of your current workplace? No or little overtime	This question is for people who answered that the workplace atmosphere is “sometimes bad” or “quite bad” in the previous question. I do not get along with my colleagues	This question is for people who answered “I feel fulfilled” or “I feel somewhat fulfilled” in the previous question. I am trusted by patients and their families	This question is for people who answered “I definitely want to” or “I would like to if I had the opportunity” in the previous question. Because it will lead to improved quality of rehabilitation	This question is for people who answered “I feel fulfilled” or “I feel somewhat fulfilled” in the previous question. I am trusted by patients and their families	Is there a sufficient number of physiotherapists and occupational therapists employed at your current workplace? Insufficient number of physiotherapists
7	2.90%	3.51%	5.39%	3.82%	2.63%	0.00%	1.81%
	What kind of stress do you feel at work? Bad workplace environment	This question is for people who answered “I would prefer not to” or “I definitely do not want to” in the previous question. Practicing rehabilitation is more rewarding for me	This question is for people who answered that the workplace atmosphere is “sometimes bad” or “quite bad” in the previous question. I may be reluctantly underrated	Objectively speaking, what are the strengths of your current workplace? Able to take paid leave as per the legal number of days	This question is for people who answered “I feel fulfilled” or “I feel somewhat fulfilled” in the previous question. I can improve my own knowledge and ability	This question is for people who answered “I definitely want to” or “I would like to if I had the opportunity” in the previous question. Because I want to develop my specialization	Do you do anything to maintain your work and health? (Multiple answers allowed) I spend time with my family
8	2.81%	3.17%	4.66%	3.66%	2.51%	0.00%	1.78%
	What do you want from management or your boss? I would like information to be promptly provided to all staff (I do not want certain people singled out to not be informed about the information)	Please indicate your current working arrangements. Day shifts	What kind of stress do you feel at work? No one to talk to	What is your specific impression of your current workplace? Human relations are good	This question is for people who answered “I definitely want to” or “I would like to if I had the opportunity” in the previous question. Because it is necessary to advance my career	What is your specific impression of your current workplace? Human relations are good	Do you do anything to maintain your work and health? (Multiple answers allowed) I take holidays
9	2.71%	3.17%	4.51%	3.66%	2.51%	0.00%	1.73%
	This question is for people who answered “I definitely want to” or “I would like to if I had the opportunity” in the previous question. Because I want to know more about rehabilitation medicine	This question is for people who answered “I would prefer not to” or “I definitely do not want to” in the previous question. I do not consider researchers to be distinguished	What is your specific impression of your current workplace? There is no future here	Do you do anything to maintain your work and health? (Multiple answers allowed) I take holidays	This question is for people who answered “I definitely want to” or “I would like to if I had the opportunity” in the previous question. Because I want to do something useful for my patients	This question is for people who answered “I definitely want to” or “I would like to if I had the opportunity” in the previous question. Because it will lead to improved quality of rehabilitation	What do you feel is the cause of your long working hours? Events and meetings are held outside working hours
10	2.61%	2.89%	3.78%	3.66%	2.44%	0.00%	1.68%
	Why did you choose your current place of work? Because the salary (base salary) is good	What is your specific impression of your current workplace? Human relations are good	What is your specific impression of your current workplace? The work is boring	Why did you quit your previous job? Please select up to five applicable answers from the following. The commute was inconvenient	This question is for people who answered “I feel fulfilled” or “I feel somewhat fulfilled” in the previous question. I can gain experience in rehabilitation for various diseases	This question is for people who answered “I feel fulfilled” or “I feel somewhat fulfilled” in the previous question. I can improve my own knowledge and ability	What do you want from management or your boss*? I would like information to be promptly provided to all staff (I do not want certain people singled out to not be informed about the information)

**Table 8:** Results of univariate linear regressions *statistically significant; t-value: t statistics; p-value: probability The results of univariate linear regression with the topic value of each respondent as the independent variable and intention to leave as the dependent variable.

	coefficient	t-value	p-value
Topic 1	1.66477	4.613	*0.000
Topic 2	-0.45017	-1.379	0.168
Topic 3	5.70979	9.409	*0.000
Topic 4	-1.02960	-2.018	*0.044
Topic 5	-1.81382	-6.945	*0.000
Topic 6	3.67213	1.101	0.271
Topic 7	0.67450	2.125	*0.034

**Table 9:** Factor analysis results The factor loadings were obtained by factor analysis of the topic items from the topic analysis and the scale responses to the single-choice questions.

	Factor 1	Factor 2	Factor 3	Factor 4	Factor 5	Factor 6	Factor 7	Factor 8
Q1	-	-	-	0.1003	-	-	-	0.8802
Q2	-	-	0.2155	-	-	-	0.1327	0.3740
Q3	-	-	-0.1526	-	-	-	-	-
Q5	-0.1236	-	0.5897	-	-	-	-	-
Q6S1	-	-	0.9254	-	-	-	-	-
Q6S2	0.1594	-	0.8695	-	-	-	-	-
Q9	-	-	-	-	-0.1020	-	-	0.5558
Q10S1	0.7993	-	0.1419	-	-	-	-	-
Q10S2	0.7834	-	-	-	-0.1499	-	-	-0.1079
Q10S3	0.4896	-	-	0.1432	-	-	-	-
Q11	0.2567	-	-0.1280	-	-	0.1277	-	0.1668
Q13	0.7424	-	-	-	-	-	-	-
Q14	0.5332	-	-	-	-	-	-	0.1075
Q18	0.4877	-	-	-	-	-0.2285	-	-
Q21	0.3245	-0.1196	-	-	0.5389	-0.1387	-	-
Q29	-	0.3098	-	-	-	0.1277	-	-
Q30	-	0.6039	-	0.1399	-	0.2029	-0.1086	-
topic1	-	-0.2090	-	-0.2531	-0.1943	-1.0636	-0.1474	-
topic2	-	1.1859	-	-0.4100	-0.1365	-	-0.1269	-
topic3	-	-0.1184	-	-0.1630	1.1204	0.1924	-	-
topic4	-	-0.1818	-	-0.1744	-	0.1233	1.0917	-
topic5	-	-0.3958	-	-0.3444	-	0.4441	-0.2419	-
topic7	-	-0.3457	-	1.2070	-0.1509	0.1695	-0.1373	-
